# Fast‐Response Variable‐Stiffness Magnetic Catheters for Minimally Invasive Surgery

**DOI:** 10.1002/advs.202305537

**Published:** 2024-01-15

**Authors:** Yegor Piskarev, Yi Sun, Matteo Righi, Quentin Boehler, Christophe Chautems, Cedric Fischer, Bradley J. Nelson, Jun Shintake, Dario Floreano

**Affiliations:** ^1^ Laboratory of Intelligent Systems, Institute of Mechanical Engineering, School of Engineering École Polytechnique Fédérale de Lausanne Lausanne 1015 Switzerland; ^2^ Multi‐Scale Robotics Lab Tannenstrasse 3 ETH Zurich Zurich 8092 Switzerland; ^3^ Shintake Research Group, School of Informatics and Engineering The University of Electro‐Communications 1‐5‐1 Chofugaoka Chofu Tokyo 182—8585 Japan

**Keywords:** active cooling, continuum robot, magnetic navigation, medical robotics, shape memory polymer, soft robotics, variable stiffness

## Abstract

In minimally invasive surgery, such as cardiac ablation, magnetically steered catheters made of variable‐stiffness materials can enable higher dexterity and higher force application to human tissue. However, the long transition time between soft and rigid states leads to a significant increase in procedure duration. Here, a fast‐response, multisegmented catheter is described for minimally invasive surgery made of variable‐stiffness thread (FRVST) that encapsulates a helical cooling channel. The rapid stiffness change in the FRVST, composed of a nontoxic shape memory polymer, is achieved by an active cooling system that pumps water through the helical channel. The FRVST displays a 66 times stiffness change and a 26 times transition enhancement compare with the noncooled version. The catheter allows for selective bending of each segment up to 127° in air and up to 76° in water under an 80 mT external magnetic field. The inner working channel can be used for cooling an ablation tip during a procedure and for information exchange via the deployment of wires or surgical tools.

## Graphical abstract

Here a magnetically steered fast‐response, multisegmented catheter is reported for minimally invasive surgery made of a fast‐response variable‐stiffness thread (FRVST) that encapsulates a helical cooling channel. The rapid stiffness change in the FRVST, which is composed of a nontoxic shape memory polymer, is achieved by an active cooling system that pumps water through the helical channel. The FRVST displays a stiffness‐change factor of 66 and a transition time 26 times faster than the noncooled version.

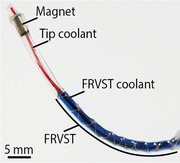

## 1 Introduction

Minimally invasive surgeries have become a popular option for treating various conditions, including cardio‐ and neurovascular diseases, as they offer a faster recovery time, shorter procedure duration, and lower risk than traditional procedures.^[^ ^1^ ^–^ ^5^ ^]^ Minimally invasive procedures frequently employ catheters,^[^ ^6^, ^7^ ^]^ which are inserted into a clearly defined anatomical conduit to undertake the intervention.^[^ ^8^ ^]^ An example of such a procedure is the treatment of cardiac arrhythmias via radiofrequency ablation, wherein a catheter is inserted in the femoral vein and guided through the right atrium of the heart.^[^ ^9^ ^]^

Conventional catheters rely on tendon‐driven actuation for navigation.^[^ ^10^ ^]^ An alternative method consists of embedding permanent magnets throughout the catheter length and controlling its movement within the body by means of an externally generated magnetic field.^[^ ^11^ ^]^ The magnetic navigation approach eliminates the need for the internal actuation mechanisms found in tendon‐driven catheters while allowing efficient navigation toward a specified body region.^[^ ^12^ ^]^ Furthermore, this method reduces radiation exposure for surgeons because they no longer need to stay with the patient during fluoroscopy and requires minimal training.^[^ ^11^, ^13^ ^]^ However, proper catheter positioning to maintain steady contact between the catheter tip and the atrial wall during cardiac ablation procedures is challenging due to continuous heartbeat activity, the complex anatomy of the human heart, the lack of 3D visualization, and the limited bending abilities of catheters.^[^ ^14^, ^15^, ^16^, ^17^, ^18^ ^]^ The latter follows from the fact that catheters driven by remote magnetic navigation systems are exposed to a unidirectional magnetic field, which limits their bending to a single direction in a particular juncture.^[^ ^8^ ^]^ To overcome this limitation, researchers, including the authors of this manuscript, have proposed segmented catheters, where each segment can change its stiffness and local responses to the magnetic field, resulting in a greater number of controllable bending directions.^[^ ^8^, ^14^, ^19^, ^20^, ^21^ ^]^

Various segmented catheter designs made of variable‐stiffness threads have been explored based on jamming technologies (fibers,^[^ ^22^ ^]^ granules,^[^ ^23^ ^]^ and layers^[^ ^24^ ^]^) and phase‐change materials (alloys^[^ ^8^, ^14^, ^20^ ^]^ and polymers^[^ ^19^, ^21^ ^]^). In jamming technologies, the transition from soft to rigid is caused by the application of negative pressure, which causes friction between internal particles. Existing catheters based on jamming technologies display a maximum stiffness change of 50 times^[^ ^22^, ^23^, ^24^ ^]^ but have a diameter between 8 and 24 mm, which is much larger than that of cardiac catheters (2.3 mm), making them unsuitable for cardiac ablation. In phase‐change materials, the stiffness can be tuned by applying electrical stimulation to increase the temperature beyond the glass transition point, thus changing the state from rigid to soft or liquid.^[^ ^25^, ^26^ ^]^ Existing phase‐change catheters display a stiffness change of 20–40 times and slower stiffness change (7‐100 s)^[^ ^14^, ^19^, ^21^, ^57^ ^]^ than jamming‐based catheters (< 1 s)^[^ ^22^ ^]^; however, phase‐change catheters can be miniaturized down to the size of standard cardiac catheters because they do not require air pressure channels, multiple fibers, and granules.

While the stiffness transition from a rigid to soft state can be fastened by tuning the resistance of a heating element and the amount of applied heat,^[^ ^19^ ^]^ the reverse transition from soft to rigid remains challenging for phase‐changing catheters. To address this problem, in the endoscopic surgery domain, which can use catheters that are up to ten times larger than those used in heart surgery, researchers have integrated an active cooling mechanism with liquid or air into catheters consisting of thermoplastic and low‐melting‐point alloys.^[^ ^27^, ^28^, ^29^ ^]^ Active cooling can decrease the reaction time by 8 times for catheters with a 15–17 mm external diameter and 25× stiffness change.^[^ ^28^, ^29^ ^]^ At the smaller scale, the preliminary study on the control strategy for endoscopic catheters of 2.5 mm external diameter showed that the active cooling can decrease the reaction time by 7 times.^[^ ^57^ ^]^ In the cardiac ablation domain, however, the integration of active cooling for fast stiffness transition into a variable‐stiffness substrate with heaters, actuators, and a working channel without sacrificing the bending performance remains challenging.^[^ ^30^ ^]^

**Figure 1:**
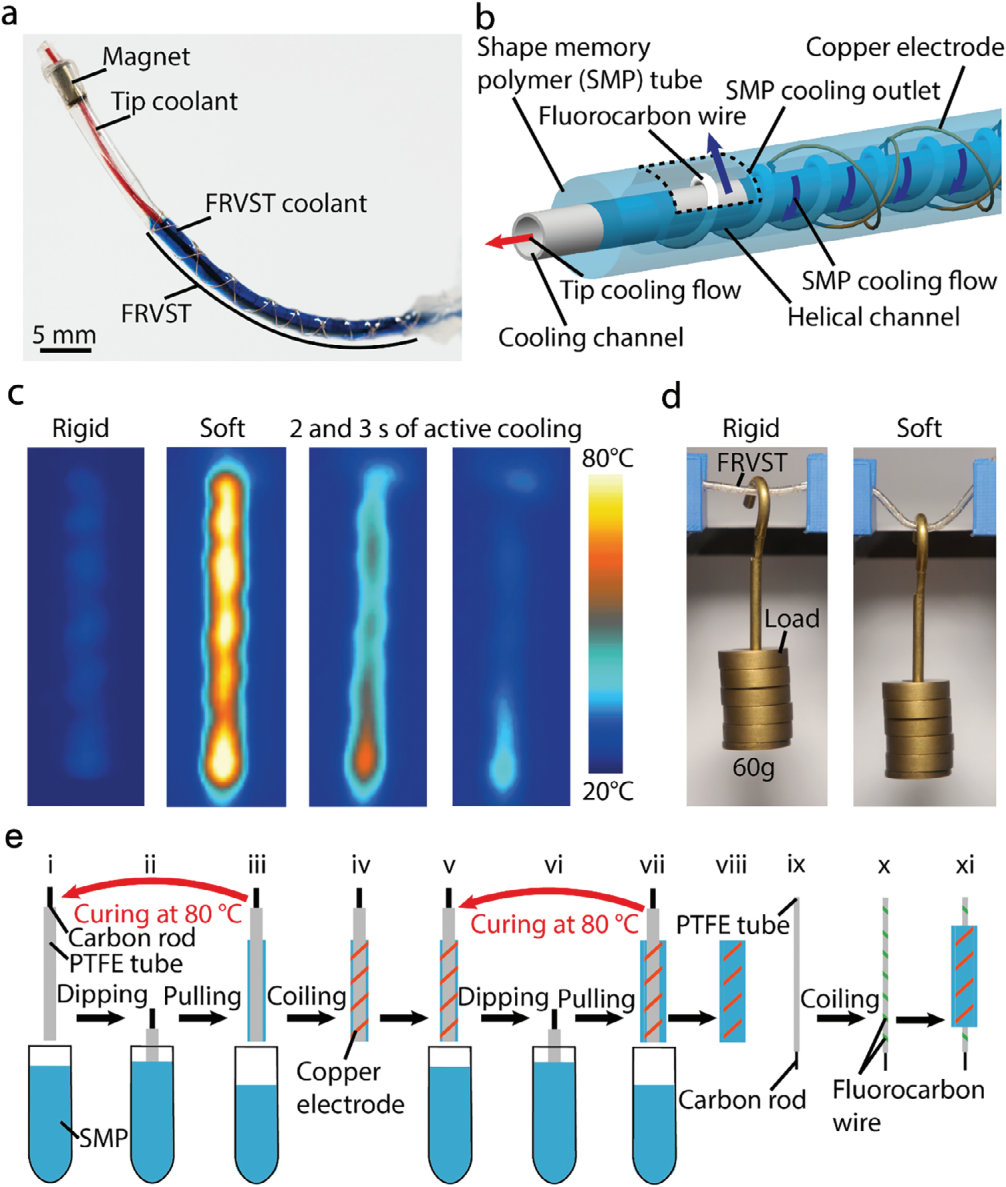
Structure, operating principle, and fabrication process of the fast‐response variable‐stiffness thread (FRVST). a) The FRVST can be integrated into a catheter for a cardiac ablation procedure. Water (in red) can be pumped through a working channel to cool down an ablation tip at the end of the catheter during surgery. Independently, water flow (in blue) can be pumped through the helical channel, enabling active cooling of the SMP layer. b) The FRVST consists of a polytetrafluoroethylene (PTFE) tube with a working channel, a helical channel made of a fluorocarbon wire, and an outer SMP tube with an encapsulated electrode used as a heater. c) The FRVST can exhibit a change in the stiffness upon indirect Joule heating of the heating electrode encapsulated into the SMP layer. d) The FRVST holds a weight of 60 g with minimal bending in the rigid state but freely bends under the same weight when heated to 80°C in the soft state. e) The fabrication process of the FRVSTs.

Here, we report a method to design and manufacture a fast‐response variable‐stiffness thread (FRVST) made of a nontoxic shape memory polymer (SMP) with an active cooling system, which is suitable for cardiac ablation (**Figure** **1**). The FRVST displays a stiffness change of 66× with a 26× faster cooling rate when the active cooling system is used. The FRVST is composed of an outer SMP tube with an encapsulated copper electrode serving as a heater to warm up the SMP tube and change its stiffness from a rigid to soft state (Figure 1a). A helical channel comprising fluorocarbon wire is placed inside the SMP tube to guide the water flow when active cooling is turned on. The helical channel is wound around an inner tube, which is used as the second independent cooling system to cool the tip of the catheter during ablation surgery (Figure 1b).

The temperature of the SMP tube increases from room temperature (25 °C) to 80 °C in 13 s via indirect Joule heating with an applied power of 2.5 W. When the applied power is turned off, it takes 115 s to naturally return to room temperature, but only 4.4 s with active cooling (0.17 L mi^−1^n cooling rate), which results in a 26‐fold transition enhancement (Figure 1c and Video S1, Supporting Information). An FRVST segment with a 2.3 mm diameter, 25 mm length, and 0.2 g weight can withstand a 60 g applied load in a rigid state but can undergo large deformation in a soft state at 40°C (Figure 1d and Video S2, Supporting Information). The FRVST exhibits a stiffness change factor (SCF) of 66 times when heated from room temperature (25 °C) to 80 °C. The FRVST is precisely fabricated by means of two automated methods: the first method is used to fabricate SMP tubes with a thickness step size of 65 µm, and the second method is used to wind fluorocarbon and copper electrodes with a 1 mm pitch size (Video S3, Supporting Information).

We validated the proposed method through the mechanical and thermal characterization of FRVSTs integrated into a single‐segment catheter. The results showed that the segment is able to bend up to 127° in air (23 °C) and up to 76° in water (36 °C) under an 80 mT external magnetic field. Then, the selective bending of a multisegmented catheter was demonstrated to achieve complicated bending curvatures in different planes. The multisegmented catheter was also tested in a 3D‐printed labyrinth to demonstrate how variable stiffness can help to avoid unnecessary contact with human tissue. Finally, the multisegmented catheter was placed in a 3D phantom of a human heart underwater at body temperature to demonstrate an ablation procedure.

## 2 Results and Discussion

We first characterized the SMP so that its material properties could be incorporated into a finite element model, which guides the design of catheters by predicting the thermal and mechanical behavior. By varying the wall thickness of the SMP tube, we performed a coupled thermoelectrical FE analysis for the FRVST to determine the heating and cooling times at different wall thicknesses. Then, by varying the wall thickness of the SMP tube, we performed a coupled thermomechancal analysis for the FRVST to determine the minimal thickness, which allows high bending in the soft state and acceptable deflection in the rigid state under external magnetic field. Based on these results, we fabricated and characterized FRVSTs in terms of wall‐thickness tunability, bending stiffness, heating‐cooling rates, and surface temperature under different conditions. We compared the results of reaction times modelling with the empirical data to validate our model. After integrating the FRVST into a single‐segment catheter, we analyzed its performance with respect to bending angle and repeatability in rigid and soft states in air and underwater. Finally, we integrated two FRVSTs with 25 mm lengths and a cylindrical permanent magnet with a 4 mm length into the tip of a multisegmented catheter with a 2.3 mm external diameter and a 0.4 mm internal diameter for the working channel. This was used in our demonstration to cool down an ablation tip during the procedure and, in tandem, enable information exchange via the implementation of wire deployment or surgical tool insertion.

### 2.1 Thermomechanical Characterization of the SMP Material

The thermomechanical behavior of the SMP material, such as the stiffness change ratio and heating/cooling time, determines FRVST performance. Therefore, it is essential to assess the thermomechanical characteristics for designing FRVSTs. We characterized the behavior of SMP and then built the model to clarify the resulting performance, which was then used to design the final device. For this purpose, a tensile test at different temperatures and dynamic mechanical analysis (DMA) of the SMP material were performed.

**Figure 2:**
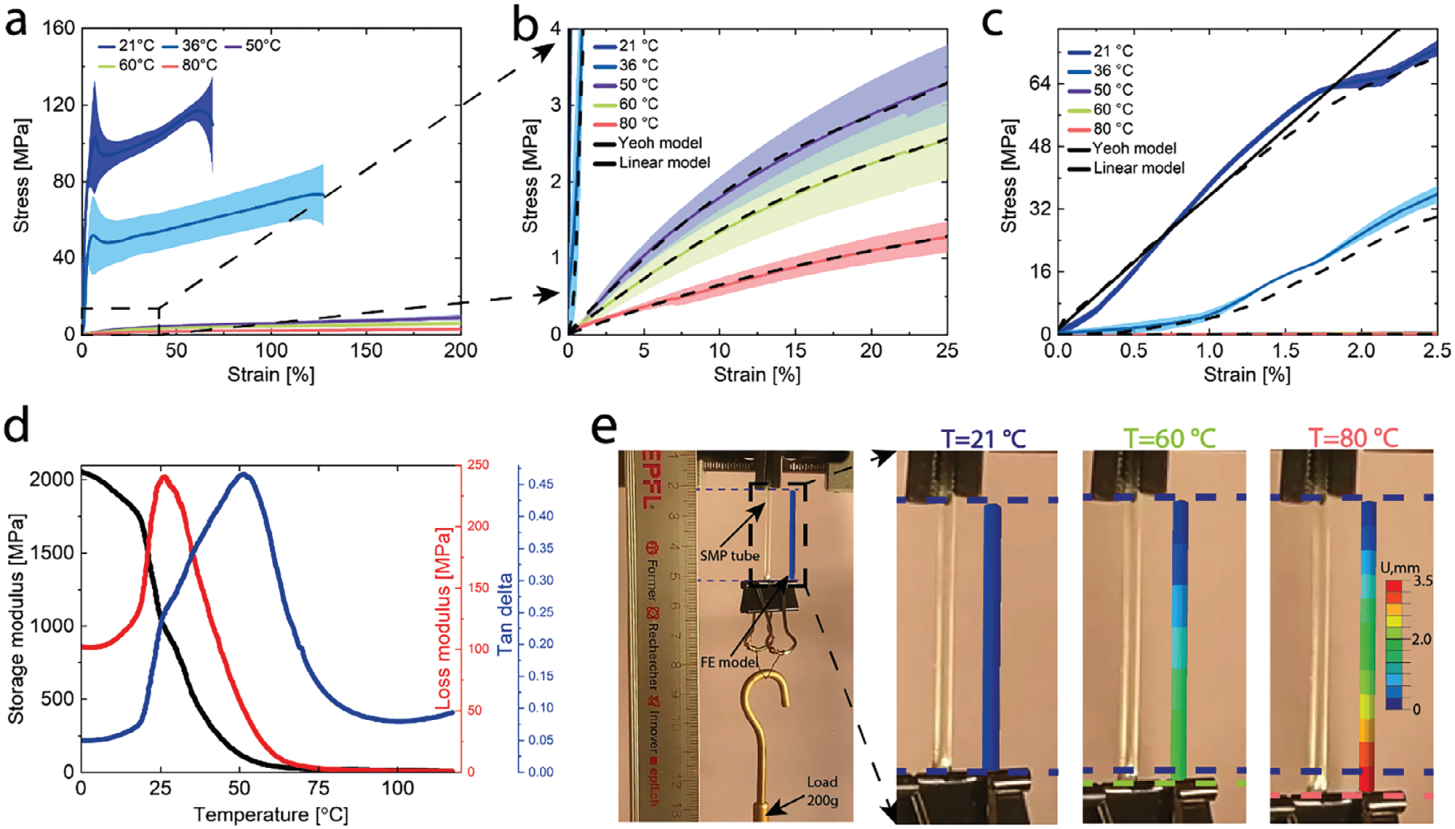
Temperature‐dependent mechanical behavior of the shape memory polymeric (SMP) material. material. a) Stress‒strain behavior of the SMP material at different temperatures. b,c) The linear and Yeoh models are well aligned with the experimental results at all temperatures. d) The storage moduli (black), loss moduli (red), and tangent delta (blue) of the SMP as the temperature was increased from 0 to 120 °C with a ramp of 3°C min^−1^. e) The SMP material model verification of the displacement at different temperatures.

We investigated the temperature‐dependent stiffness variation of the SMP by conducting uniaxial tensile tests via a tensile testing machine (Instron 5965) at a constant speed of 50 mm/min until the specimen fractured or exceeded 200% strain (**Figure** **2**a; Figure S3 in the Supporting Information). As a result, the SMP exhibits a linear stress–strain behavior with a modulus of 3.4 GPa at a room temperature of 25 °C. The stiffness dramatically decreases to 640 and 5.6 MPa at 36 and 80 °C, respectively, followed by a modeled nonlinear stress–strain behavior (Figure 2b,c). These results indicate an SMP stiffness change similar to other phase‐changing materials used in variable‐stiffness catheters, making it a suitable candidate for this study.^[^ ^14^, ^19^, ^21^ ^]^ The Yeoh model was chosen because it provides a good fit with the SMP data in the soft state and has been previously used to model polyurethane elastomers for biomedical applications (Section S1 in the Supporting Information).^[^ ^31^, ^32^ ^]^ For our thermomechanical model, we decided to use a linear model for a rigid state (21 °C) and a Yeoh model for SMP temperatures of 36, 50, 60, and 80 °C because they provide the closest fit at these temperatures (Figure S4 in the Supporting Information).

To determine the stiffness variation of the SMP material at different temperatures, we evaluated the thermomechanical behavior of MM5520 by performing a dynamic mechanical analysis (DMA) test by heating the sample from 0–120 °C with a ramp of 3 °C min^−1^ under tension (Figure 2d). The glass transition temperature (Tg) was determined as the temperature at which the tanδ (the ratio of the loss modulus (in red) to the storage modulus (in black)) curve reaches its maximum and is equal to 52 °C.^[^ ^33^ ^]^ The increase in temperature from 0–120 °C resulted in a 200‐fold decrease in stiffness. The current material reaches a tanδ pike of 0.48, which is larger than that of carbon‐infused SMP (0.28).^[^ ^19^ ^]^ As discussed for polymer–carbon nanotube interactions, the energy dissipation decreases with increasing carbon loading, leading to a decrease in the tanδ peak height.^[^ ^34^ ^]^ The tanδ pike of the current SMP is wider than in the case of pure and carbon‐infused SMPs used in soft robotics applications,^[^ ^19^, ^35^ ^]^ revealing higher interfacial interactions that are typical for some polyurethane (PU) polymers.^[^ ^34^, ^36^ ^]^ The stiffness variation and absolute values observed from the uniaxial tensile tests agree well with the results from the DMA tests.

We performed the finite‐element (FE) simulation of the SMP tube using ABAQUS (V6.14, Dassault Systèmes Simulia Corp., USA) and then compared the results with an experiment (Figure 2e). The boundary conditions for the thermomechanical model were consistent with the experiment (Section S2 in the Supporting Information). The model accurately predicts an elongation of the rigid and soft SMP tubes with a length, inner and outer diameters of 30, 0.7, and 2.2 mm under a dead load of 0.2 kg. The modeled elongations (1.9 and 3.3 mm) and experimental elongations (2 and 3.2 mm) at 60 and 80 °C were observed.

### 2.2 Fabrication Method of FRVST: Description and Characterization

The base material for our FRVSTs is a polyurethane‐based SMP (SMP Technologies Inc., MM5520). We chose an SMP material because SMPs are widely used in biomedical applications such as stents, drug delivery, and bone tissue engineering^[^ ^3^, ^37^, ^38^ ^]^ due to their thermoplastic properties (ability to change the stiffness under applied thermal stimuli), low cost, high recoverable strain levels (300% vs 10% compared to other programmable materials such as shape memory alloys), and ease of manufacturing, which allows the fabrication of scalable and complex soft medical devices.^[^ ^19^, ^39^, ^40^ ^]^ Compared to MM4520 used in a previous study,^[^ ^19^ ^]^ MM5520 demonstrates excellent biocompatibility attributes defined by in vitro cytotoxicity, cytocompatibility, inflammation, thrombogenesis, and platelet adhesion tests.^[^ ^39^, ^41^ ^]^ All the aforementioned results were obtained within a time duration surpassing the length of a cardiac ablation procedure (284 min), making MM5520 a suitable material candidate for minimally invasive devices.^[^ ^42^ ^]^ However, since its thermomechanical behavior is not discussed in the literature, in this study, we also perform material characterization.

The FRVST fabrication procedure starts with the formation of an SMP layer on the PTFE tube by means of our previously described dipping method (for details, see^[^ ^19^ ^]^). In this method, a PTFE tube with an external diameter of 1.4 mm is fixed on a carbon rod and dipped vertically into the SMP mixture. Curing in an oven induces the formation of an SMP layer on the tube (Figure 1e‐i,ii). This dipping step can be repeated multiple times to achieve the desired thickness of the SMP layer (Figure 1e‐iii). However, that previously described method relied on manual dipping without precise control of retraction and spin speeds of the PTFE tube with an SMP layer, resulting in poor thickness control. Here, we address the problem by developing an automated dipping setup (Figure S1 in the Supporting Information), which allows precise definition of SMP thickness by controlling the number of dips, extraction, and spin speeds.

After two dipping steps, a copper electrode wire is wound around the SMP tube using an automated winding machine (Figure 1e‐iv and Figure S2 in the Supporting Information). This wire will be used to heat up the SMP layer during catheter operation. The wired SMP tube is then dipped again in the SMP mixture two times to encapsulate the copper electrode and attain the intended thickness (Figure 1e‐v‐vii). Finally, the fabricated SMP tube is removed from the PTFE tube (Figure 1e‐viii) and placed aside. To form a cooling system inside the FRVST, we selected a new thin PTFE tube with an external diameter of 0.6 mm and a working channel of 0.48 mm, which matches well as a lumen for cardiac ablation catheters. Then, a fluorocarbon wire is wound around the PTFE tube to form a helical channel, which will be used as an active cooling mechanism to guide the water flow through the catheter (Figure 1e‐ix,x).^[^ ^43^ ^]^ As the final assembly step, the fabricated SMP tube is slipped onto a working channel tube with a helical channel (Figure 1e‐xi).

**Figure 3:**
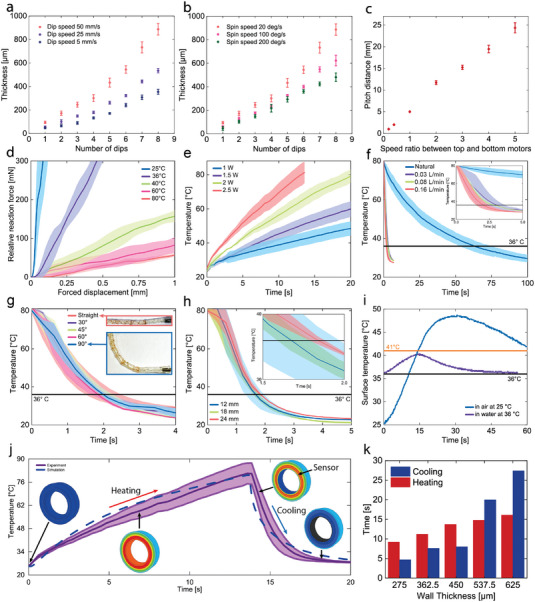
Fast‐response variable‐stiffness thread (FRVST) characterization. The coating thickness of the FRVSTs was 0.45 mm. Three measurements were collected for three different samples in all the tests. a) Thickness of the shape memory polymer (SMP) layer as a function of the dip speed and number of dips. b). Thickness as a function of spin speed. c) The pitch distance between two turns of heating or fluorocarbon wires as a function of the speed ratio between the winding machine's motors. d) Reaction force of the FRVST against forced displacement under a three‐point flexural test at different temperatures. e) Heating rate of the FRVST from room temperature (25 °C) for different applied powers. f) Cooling rate of the FRVST from 80 °C to room temperature for different cooling rates. g) Cooling rate of the FRVST from 80 °C to room temperature for different bending angles. h) Cooling rate of the FRVST for different cooling channel designs. i) FRVST with the SMP layer heated to 80 °C, reaching a surface temperature of 49 °C in air and 40.3 °C in water at 37 °C without flow. j) Comparison of the simulated and measured heating‐cooling cycles of the FRVST. k) Simulated heating and cooling times for the FRVST with different SMP wall thicknesses.

The FRVST in this study is fabricated through an automated setup where a PTFE tube is dipped into a mixture of SMPs (Video S3, Supporting Information). After extraction, the PTFE tube starts spinning to form an even layer of the SMP coating on the surface of the PTFE tube. The resulting thickness of the SMP layer depends on the number of dips and spinning speed. As shown in **Figure** **3a**, the thickness of the SMP layer increases from 40, 50, and 90 to 355, 535, and 890 µm from the first to the eighth dip for a spin speed of 20 deg s^−1^ and dip speeds of 5, 25, and 50 mm ^−1^s, with maximum step sizes of 70, 90, and 130 µm, respectively. For spin speeds of 20, 100, and 200 deg s^−1^, the thickness of the SMP layer increases from 90, 60, and 45 to 890, 620, and 480 µm from the first to the eighth dip at a dip speed of 50 mm ^−1^s, with maximum step sizes of 130, 75, and 65 µm, respectively (Figure 3b). Extraction speed equals to the dip speed within the same experiment. All data sets show the same behavior for all speeds, which coincides with the literature, suggesting that the thickness is highly controllable.^[^ ^44^ ^]^ Ten times higher dip and 10 times lower spin speeds allow fabrication of a 2.5 times thicker SMP coating, which results in a 2 times higher standard deviation.

Then, we characterized the fabrication of a helical channel using an on‐custom‐made winding setup (Figure 3c; Figure S2 in the Supporting Information). The generated pitch distance between two windings of a fluorocarbon wire depends on the speed ratio between the top and bottom motors of the setup. The pitch distance increases linearly from 1 to 25 mm under speed ratios from 0.2 to 5.

### 2.3 Thermomechanical Characterization of The FRVST

Before assembling a single‐segment catheter, we performed thermomechanical characterization of the FRVST to ensure that its characteristics matched the requirements for cardiac catheters. We started by assessing the variable‐stiffness performance of the FRVST by characterizing the bending stiffness through a three‐point flexural test, where the FRVST was placed in a universal testing machine equipped with a temperature box. The test was conducted at five temperatures: 20 °C for the rigid state, and a human body temperature of 36 °C, and 40, 60, and 80 °C for the soft states. During the test, the deflection and reaction force of the FRVST were measured. The results of three measurements for three devices are plotted In Figure 3d. Based on the Eule—Bernoulli beam theory, boundary conditions, and geometrical parameters, the SCFs of the FRVST between the rigid state (20 °C) and 36, 40, 60, and 80 °C were found to be 5.6, 23.4, 45.2, and 66.1, respectively (Section S3 in the Supporting Information).

We then investigated the effect of the applied power on the heating rate by measuring the temperature of the FRVST under 1, 1.5, 2, and 2.5 W (Figure 3e) to characterize the heating time. The FRVST can be heated in air from 25 °C (room temperature) to 80°C in 25 and 13 s under 2 and 2.5 W, respectively. Increasing the current can shorten the heating time, but the impact is restricted since the diffusion between the heating wire and the SMP layer requires time. Moreover, further amplifying the current may lead to circuit burnout from overheating.

The FRVST utilizes the helical open loop cooling system to greatly enhance the cooling speed, whereas existing FRVSTs for cardiac ablation devices rely on slow convection cooling.^[^ ^8^, ^14^, ^19^, ^21^ ^]^ We measured the cooling rate of the FRVST under natural convection and different flow rates equal to 0.03, 0.08, and 0.17 L mi^−1^n, which are in the same range as currently utilized for cardiac ablation surgeries.^[^ ^45^ ^]^ The water temperature was equal to room temperature. The FRVST was heated to 80 °C under 2 W, and after reaching the desired temperature, the heat was turned off, and active cooling was turned on. Active cooling was performed using an automated syringe pump (Figure S5 in the Supporting Information). Compared with passive cooling, which requires ≈115 s, using water coolant at 0.03, 0.08, and 0.17 L mi^−1^n takes only 6.6, 6.3, and 4.4 s to decrease the temperature down to 28 °C, which improves the cooling rate by 17, 18, and 26 times, respectively (Figure 3f). To achieve the temperature of a human body (36 °C), it takes 46, 3, 2.65, and 1.8 s under natural conditions, and coolant rates of 0.03, 0.08, and 0.17 L mi^−1^n, therefore enhancing the cooling rate by 15, 17, and 25 times, respectively.

The use of the FRVST in minimally invasive devices requires an ability to bend up to 90° without causing a change in the cross section to execute cooling at the same cooling rate. Thus, a test to measure the cooling speed at bending angles of 30, 45, 60, and 90° was performed (Figure 3g). The room‐temperature water was pumped through the helical channel at a rate of 0.17 L mi^−1^n. The FRVST cools from 80 °C to a human body temperature of 36 °C in the straight and bent configurations in 1.8 and 1.9 s, which indicates the good performance of the cooling system in the bent state.

The dimensions of the cooling channel can be a parameter that determines the cooling rate of the FRVST. To clarify the influence of the channel dimensions, we prepared three FRVSTs with cooling channel pitch distances of 12, 18, and 24 mm. They were evaluated to clarify the effect of the helical channel on the cooling performance (Figure 3h). The FRVSTs with helical channel step sizes of 12 and 24 mm cool from 80 °C to a human body temperature of 36 °C in 1.65 and 1.8 s, respectively. Using a 12 mm helical channel results in a 0.15 s shorter cooling time but requires twice the length of the cooling wire, which makes the FRVST stiffer and reduces the SCF between the rigid and soft states. As a result, a 24 mm helical channel was used in the study.

When integrated in a minimally invasive device such as a catheter, the surface temperature of the FRVST must stay in a biocompatible temperature range (below 41 °C) to ensure safe operation inside the body. Thus, we determined the maximum temperature of the outer SMP layer in the soft state by characterizing the surface temperature in air at room temperature (25 °C) and in water at 36 °C without forced flow. The water temperature was set to replicate the thermal characteristics of a device in the human body, as both have comparable heat‐transfer properties.^[^ ^8^ ^]^ A thermistor was glued in the middle of the FRVST surface and was isolated against the water with a layer of glue. The surface temperature was measured by thermistors when heat was applied. In addition to the copper electrode used for heating, we wound the second copper electrode closer to the surface of the SMP layer. This electrode was used to control the stiffness of the FRVST by measuring its resistance, which changes at different temperatures of the SMP layer (Figure S6 in the Supporting Information). The use of a copper wire as a sensor was already utilized to control the state of phase‐change materials with a precision of 0.2 °C.^[^ ^20^, ^21^ ^]^

First, the FRVST was heated to 80 °C in air with an applied power of 2 W (applied voltage 2 V). The temperature was controlled using a thermal camera and by monitoring the resistance change in the sensing wire (Figure S6 in the Supporting Information). When submerged in water, the applied power was 4.5 W, which corresponds to an applied voltage of 3 V. The power was turned off when a sensing wire relative resistance change of 20% was achieved (Figure 3i). This value corresponds to the resistance change of the sensing wire when the SMP body is heated to 80 °C.The surface temperature of the FRVST reached 49 °C in air and 40.3 °C in water when the SMP body was heated to 80 °C. These results indicate that the FRVST can be operated under a biocompatible temperature range of 41 °C.^[^ ^46^ ^]^ During minimally invasive surgery, the FRVST will be exposed to fluid flow, which increases the heat dissipation and lowers the surface temperature even more. The surface temperature differs from the FRVST body temperature because of the well‐studied thermal gradient caused by the heating system and the environment.^[^ ^20^ ^]^ The encapsulation of the FRVST consists of an SMP material, whose thermal conductivity of 210 W mm^−1^*K is one order of magnitude lower than that of silicone with a thermal conductivity of 2730 W mm^−1^*K used in previous studies.^[^ ^14^, ^19^ ^]^

The FRVST stiffness change factor can be tuned by changing the thickness of the SMP layer. Thus, we performed a thermal‐electrical simulation of the heating‐cooling cycle using ABAQUS to later understand how the heating and cooling durations are affected by the increase in layer thickness (Section S3 in the Supporting Information). The information regarding the material properties of the SMP is limited; thus, we determined the material constants of the SMP needed for the simulation, including density, specific heat capacity, thermal conductivity, and electrical conductivity (Section S4,S5 and Figure S7 in the Supporting Information). The simulated and measured temperature‒time relationships during a heating‐cooling cycle showed a good fit (Figure 3j). The measured heating‐cooling cycle was performed under 2.5 W of power in the heating phase and under a cooling rate of 0.17 L mi^−1^n in the cooling phase. With the help of this verified model, additional projections were made about the heating and cooling process of the FRVST when the thickness of its SMP layer varies (Figure 3k). If the thickness of the SMP layer increases from 0.275 to 0.625 mm, then the duration of heating increases from 9 to 16 s, and the duration of cooling increases from 4.7 to 27.4 s. Later in the paper, a trade‐off between reaction times and bending stiffness in the rigid and soft states at different SMP layer thicknesses is discussed.

### 2.4 Characterization of a Single‐Segment FRVST Catheter

**Figure 4:**
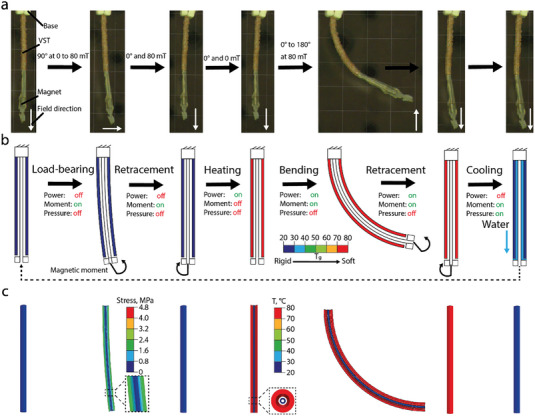
Working principle of the single‐segment catheter with the fast‐response variable‐stiffness thread (FRVST). a) Images of the working cycle under an applied external magnetic field. b) Working principle of the standard working cycle of the single‐segment catheter. The catheter can withstand an applied magnetic moment in the rigid state during the load‐bearing phase. In the soft state, it bends when the magnetic field is applied. c) FE simulation results in a typical working cycle. The boundary conditions for the simulation are the same as in the experiment.

After characterizing the FRVST, we integrated it into the single‐segment catheter, the design and working cycle of which are illustrated in **Figure** **4**a. It consists of an FRVST attached to a base on the top end and is equipped with a cylindrical permanent magnet with a 4 mm length and 2 mm external diameter at the tip. The catheter has a 55 mm length, a 2.3 mm outer diameter, and a 0.48 mm working channel. The permanent magnet is inserted and glued inside the SMP tube. The working channel passes through the lumen inside the cylindrical magnet and can be used to deliver coolant to the tip of the catheter, for example, during an ablation procedure. The active cooling system of the SMP layer, cooling system of the tip, heating, and sensing wires are plugged into the power supply and multimeter at the base to deliver power and control the state of the SMP layer. The catheter was designed with the same size as existing cardiac ablation catheters.^[^ ^43^ ^]^

A working principle of the single‐segment FRVST catheter is illustrated in Figure 4b. In the rigid state, when power is not applied, the catheter can withstand an applied magnetic torque generated by an external magnetic field from 0 to 80 mT. When the power is applied and the SMP layer becomes soft, the catheter can be freely bent in the direction dictated by the magnetic torque from 0 to 180° at 80 mT. Once the shape of the catheter is fixed, the cooling system is turned on, and the SMP layer becomes rigid. To further investigate the actuation capabilities of the single‐segment catheter in the rigid and soft states, we performed an FE simulation by implementing the Yeoh hyperelastic model into the SMP layer of the catheter. (Section S2 in the Supporting Information). Figure 4c depicts that the FE simulation accurately matches the experiment. During the loading‐carrying stage, both the experiment and simulation show a small catheter deformation. Furthermore, the simulation indicates that the SMP layer bears the majority of the load and that there is negligible stress on the soft section of the catheter.

**Figure 5:**
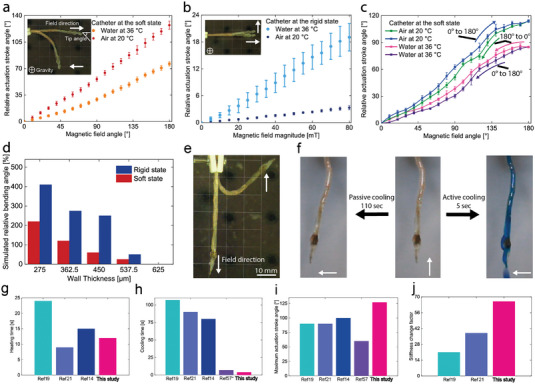
Characterization results of the single‐segment catheter with a fast‐response variable‐stiffness thread (FRVST). a) Actuation stroke angle as a function of the magnetic field angle in the soft state. b) Actuation stroke angle as a function of the magnetic field magnitude in the rigid state. c) Actuation stroke angle as a function of the magnetic field angle in the soft state. The values represent three tested catheters and five cycles per device. d) Simulated actuation stroke angle as a function of the SMP wall thickness in the rigid and soft states. e) Bending actuation of the FRVST single‐segment catheter in the soft state. f) Demonstration of the active cooling of the single‐segment catheter. g–j) Comparison of heating‐cooling times, maximum actuation stroke angle, and stiffness change factor with relevant works in the literature.

We evaluated how well a single segment of a FRVST catheter performs by examining its bending angle in both the soft and rigid states in the air and water when exposed to external magnetic fields by a hospital‐compliant electromagnetic navigation system (eMNS). The bending angle in the soft state determines how flexible the catheter is, affecting its dexterity. Conversely, in the FRVST rigid state, the bending angle determines the magnetic torque that the catheter can resist, thereby determining the level of shape fixity that can be achieved. A permanent magnet with a dipole moment *m* at position *p* under external magnetic field *B* generates a magnetic torque *T_m_* equal to *T_m_* (*p*) =  *m*  × *B*(*p*).^[^ ^19^ ^]^ When the magnetic field is perpendicular to the dipole moment, the maximum magnetic torque is attained, while the torque is minimal when they are aligned. However, selecting permanent magnets with higher dipole moments or different volumes allows for the magnetic torque to be adjusted. First, we characterized the deflection of a single‐segmented catheter in a soft state by varying the magnetic field angle from 0 to 180° in air at 20 °C and water at a human body temperature of 36 °C (**Figure** **5a**). The catheter can bend up to 127° and 76° in the air and water under a magnetic field magnitude of 80 mT. In the rigid state, the catheter can withstand the applied magnetic moment of 80 mT perpendicular to the catheter direction and bend only up to 3.3° and 19° in the air and water, respectively (Figure 5b). Note that the values in Figure 5a and b represent three tested catheters and three cycles per device. In the soft state, the device also demonstrates high repeatability with a low hysteresis for five cycles in both air and water (Figure 5c).

We used the FE thermomechanical model discussed previously to investigate the influences of the SMP layer thickness on the resulting bending performance in the rigid and soft states in air (Figure 5d). The simulated results indicate that by decreasing the SMP layer thickness from 0.625 to 0.275 mm, an increase of four and two times in the bending angle in the rigid and soft states can be achieved. Therefore, reducing the catheter's wall thickness from 0.625 mm to 0.275 mm results in a heating‐cooling cycle that is 2.6 times faster but comes at the cost of a 4 times decrease in load‐bearing capabilities.

The catheter can bend up to 130° in the desired direction in the soft state under 80 mT (Figure 5e). We demonstrated the soft‐to‐stiff state transition by cooling down the catheter with and without active cooling while the catheter was rotated by applying a rotated magnetic field from 0 to 180°. The catheter changes the state from soft to rigid in 110 and 5 s with passive and active cooling (Figure 5f). The motion and stiffness change rate of the device can be observed in Video S4, Supporting Information. Compared with other variable‐stiffness catheters with passive cooling in the literature, our catheter has a comparable heating rate, 20× faster cooling rate from 80 down to 25 °C, 30° larger actuation stroke angle, and, at the same time, 1.7× higher SCF (Figure 5g–j).^[^ ^8^, ^14^, ^21^ ^]^ Compared with the smallest endoscopic variable stiffness catheter with active cooling system, our catheter has a 2× larger actuation stroke angle and 7× faster cooling rate from 51 down to 37 °C.^[^ ^57^ ^]^ Moreover, multiple FRVSTs can be integrated into the same catheter body to achieve more dexterous positioning of the catheter's tip.

### 2.5 Multisegmented Catheter for Cardiac Ablation

**Figure 6:**
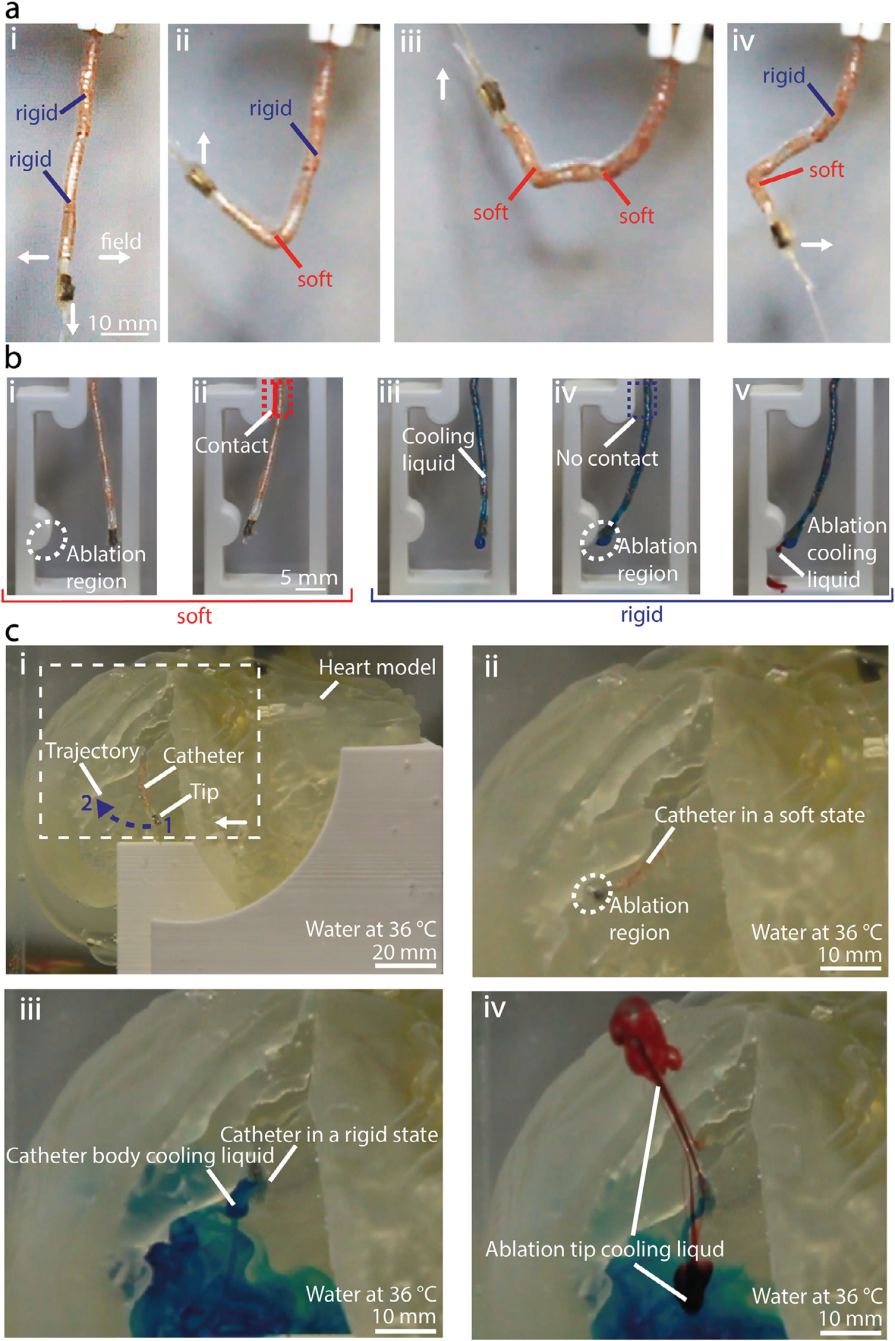
Multisegmented catheter design, performance, and application. a) The catheter consists of a fast‐res variable‐stiffness thread (FRVST) with two heating coils winded in series that allow an independent stiffness change in two segments formed in the same FRVST. The catheter is placed in an electromagnetic navigation system (eMNS) i) when both FRVSTs are solid and ii–iv) when one of them is successively soft. b) A demonstration of the variable‐stiffness capabilities of the multisegmented catheter. i) The catheter can be navigated to a specific ablation point by an external magnetic field. ii) In the soft state, it cannot hold the shape and avoid physical contact. iii) After cooling, iv) the stiff catheter can be accurately navigated to a specific region without applying any pressure on other regions. v) The working channel can be used to cool an ablation tool independently from the cooling of the FRVST. c) A demonstration of catheter application in the cardiac ablation procedure. The demonstration is performed underwater on a 3D phantom of the human heart placed in an eMNS. i) The catheter is magnetically steered from the point 1 to the point 2. ii) After reaching the ablation region, iii) blue cooling liquid is pumped through the helical channel to cool down the segments and lock the shape of the catheter. iv) Then, the red cooling liquid is pumped through the working channel to demonstrate the cooling process at the interface between the ablation tip and human tissue.

Using the previously described method and materials, we developed a multisegmented catheter with two independently controlled segments and demonstrated its use for cardiac ablation in a 3D‐printed model of the human heart at body temperature (**Figure** **6**). The catheter consists of a permanent magnet at the tip, a working channel, which can be used to cool an ablation tip during the surgery, and two heating coils that can independently heat a part of the FRVST (Figures 1 and 6a). Each of the heating coils covers 25 mm of the FRVST length with a total length of 75 mm. The device structure was encapsulated by dipping it into the SMP material.

The catheter was first placed in an aquarium in the middle of the eMNS working area. The magnetic field was applied perpendicular to the length of the catheter to demonstrate the deflection of the tip in the rigid state (Figure 6a‐i). Then, the FRVST was locally heated by applying power to one of two heating coils and bent to realize catheter deflection. The selective activation of heating coils of the FRVST enables different deformations of the multisegmented catheter (Figure 6a‐ii,iii). Each of the segments of the catheter can be flexed up to an angle of 90 degrees, while the second segment can resist an applied magnetic torque (Video S5, Supporting Information). The catheter is capable of producing complex curvatures in different planes by inducing sequential stiffening and softening of individual segments (Figure 6a‐iv). Consequently, the catheter is endowed with greater dexterity than a single‐segment catheter, owing to the additional degrees of freedom afforded by the integration of two heaters in the FRVST. When both segments are in a rigid state, the workspace of the catheter consists of a circular trajectory with a diameter of 7 mm. Reduced stiffening of the segments increases the workspace by 28 mm when the heater closer to the permanent magnet is heated and by 42 mm when the bottom heater closer to the base is activated.

To illustrate the potential use case of the catheter in medical applications, the 3D‐printed labyrinth was placed in the aquarium under the catheter (Figure 6b). In the soft state, the catheter cannot avoid contact with the channel walls while being steered to the potential ablation region (Figure 6b‐i,ii). This contact causes frictional forces by the catheter on the blood vessel wall, which can lead to vasoconstriction, injury, and complications such as reactive intimal proliferation or distal embolization, potentially resulting in end‐organ ischemia and infarction.^[^ ^60^, ^61^ ^]^ However, by changing the state of the top segment from soft to rigid through active cooling (blue liquid), an ablation region can be achieved without any contact with the channel walls (Figure 6b‐iii,iv). The catheter remains in its position while the demonstration of an ablation procedure is shown with the red ablation cooling liquid (Figure 6b‐v and Video S6, Supporting Information).

Finally, to demonstrate the catheter's navigation capability in reaching ablation target regions within the heart chambers, it was guided through a 3D‐printed model of a human heart submerged underwater at body temperature (Figure 6c‐i and Video S7, Supporting Information). The device was placed in the left ventricle and then steered, being in the soft state, from the initial point 1 to the final point 2 to the ablation region as it is done during a real surgery (Figure 6c‐i,ii).^[^ ^47^ ^]^ The FRVST inside the catheter was cooled to lock the shape by pumping cooling liquid (in blue) through the helical channel (Figure 6c‐iii). Finally, we demonstrated the use of a working channel to pump coolant (in red) to lower the temperature at the interface between the human tissue and the ablation tip (Figure 6c‐iv). The motion of the multisegmented catheter in a 3D‐printed model of the human heart can be observed in Video S7 (Supporting Information).

## 3 Conclusion

We have described a method for designing fast‐response and stiffness‐tunable variable‐stiffness devices made of shape memory polymers (SMPs), namely, variable‐stiffness threads (FRVSTs). When heated from 25 to 80 °C, the current design can provide a 66× stiffness change. We integrated a helical channel in the FRVST design that enables open irrigated active cooling of the nontoxic SMP layer, which decreases cooling times by 26× from the soft to the rigid state compared to passive cooling. We performed material characterization of the SMP and developed an FE thermomechanical model to discuss the trade‐offs among cooling/heating rates, bending performance, and SMP wall thickness. Fabrication of the FRVST utilizes a dipping and winding technique to produce SMP layers and helical channels with a predetermined thickness and pitch distance, respectively. Using this technique, we developed single‐segment and multisegmented FRVST catheters with integrated permanent magnets. We demonstrated selective bending of each of the segments by controlling the stiffness of each of the segments in the hospital‐compliant electromagnetic navigation system (eMNS). In the soft state, the catheter can bend up to 127° and 76° in air and water under a magnetic field magnitude of 80 mT. When switched to the rigid state, the catheter can withstand applied magnetic fields up to 80 mT and bend only up to 3.3° and 19° in the air and water. An integrated active cooling mechanism allows the shortening of the stiffening process from 117 to 4.4 s. Our catheter design has a 7× faster cooling rate, 30° larger actuation stroke angle, and, at the same time, 1.7× higher stiffness change factor (SCF) compared to existing proof‐of‐concept multisegmented variable stiffness catheters for cardiac ablation.^[^ ^8^, ^14^, ^19^, ^57^ ^]^

While variable‐stiffness catheters with previous designs can simplify tip positioning and increase applied force,^[^ ^14^, ^20^ ^]^ they may also prolong the procedure duration because of a long 90‐second heating‐cooling cycle.^[^ ^19^ ^]^ The current design with a heating‐cooling cycle of 17 s can significantly decrease the duration of surgery, making variable‐stiffness catheters more feasible for practical use.

In addition to its potential utility in minimally invasive devices across various medical procedures, the FRVST holds promise for incorporation into smart fabrics, where it could be woven into textiles.^[^ ^48^ ^]^ Compared to previously developed phase change material‐based variable‐stiffness threads, our FRVST can change its state faster without limiting the motion of the user.^[^ ^25^, ^49^ ^]^ Another potential application is flying vehicles, where the rapid state change in our FRVSTs can enable faster morphing between different locomotion modes.^[^ ^26^ ^]^

## 4 Experimental Section

### Tensile Test Sample Fabrication

The SMP mixture was prepared by dissolving 10 g of pellets of a shape memory polyurethane (SMP Technologies, MM 5520) in 60 g dimethylformamide (DMF) for 8 h via a magnetic stirrer at 60 °C. The SMP mixture was poured onto a glass substrate on an automated film applicator coater (ZAA 2000‐Zehntner‐Automatic film applicator). The thickness of the gap height of the applicator was adjusted to 700 µm, and the mixture was spread at a drawing speed of 2 mm ^−1^s. Afterward, the SMP mixture on the glass substrate was placed in an oven for a minimum of 8 h at 80 °C to evaporate excess DMF. The same procedure was followed for the second and third layers of the SMP with an increase in the gap height by 50 µm at every step to account for the thickness of the previous layer. The sheet was delaminated from the glass substrate using a sharp razor blade. The specimens were then cut from the SMP sheet according to ISO standard 527‐3 for the determination of tensile properties of plastic films and sheets (Figure S3 in the Supporting Information).

### Tensile Test of the SMP Samples

Tensile tests were conducted to obtain the stress‒strain relationships of the SMP at room temperature (25 °C), human body temperature (36 °C), 50, 60, and 80 °C. A tensile testing machine (Instron 5965) equipped with a 500 N load cell and a thermal chamber was used. A sample with a dog‐bone shape and a thickness of 0.15 mm was moved at a constant speed of 50 mm min^−1^ until the specimen fractured or exceeded 200% strain (Figure S3a, Supporting Information). The collected load and elongation data were converted to a stress‒strain curve to obtain Young's modulus by applying a linear or Yeoh model. The elongation and width changes before and after the tensile test were measured using a caliper and micrometer to determine the Poisson's ratio µ of the SMP using the following formula for large deformations:

(1) $$\begin{array}{c} -\frac{h_{final}-h_{0}}{h_{0}}=1-{\left(1+\frac{l_{final}-l_{0}}{l_{0}}\right)}^{-\nu } \end{array}$$

where *h*_0_, *h_final_*, *l*_0_, and *l_final_* were the initial, final width, initial, and final length of the dog‐bone sample, respectively. The determined Poisson's ratio ν of the SMP was equal to 0.48. Thus, the material was modeled as incompressible.

### Dynamic Mechanical Analysis (DMA) of the SMP Material

The DMA test for the SMP MM5520 was performed in the tension film mode on three strip samples (dimension 30 mm × 5 mm × 0.2 mm) fabricated via the layer deposition technique with a dynamic mechanical analyzer (TA Instruments DMA Q800). The sample was heated from 0—120 °C with a ramp of 3 °C min^−1^ under tension. Initially, the sample was cooled to 0 °C and stabilized for 5 min to reach thermal equilibrium. The strain oscillated at a frequency of 1 Hz with a peak‐to‐peak amplitude of 0.2% of strain.

### Helical Channel Design Characterization Using an On‐Custom‐Made Winding Machine

An on‐custom‐made winding machine consists of two step motors located along the same axis with drilling chucks at each of the tips serving as grippers (Figure S2 in the Supporting Information). An SMP or PTFE tube was inserted between the motors and held by chucks in the horizontal position. The motors provide rotation of the tube. A coil with a winded wire (a copper or a fluorocarbon wire) stays on the linear stage, which was located under the motors. The linear stage provides movement of the coil along the length of the held SMP or PTFE tube. The control of step motors and the linear stage was performed using an Arduino Uno and step motor drivers in the control box. The rotation speed of the top step motors was fixed. The linear speed of the linear stage varied to provide a different pitch of the helical channel. Thus, the speed ratio between the two top motors and the bottom motor of a linear stage varied in the experiment. The experiment was conducted three times for three SMP tubes under speed ratio coefficients of 0.2, 0.4, 1, 2, 3, 4, and 5.

### Automated Dipping Setup Design

The automated dipping setup has three degrees of freedom: one degree in translation to perform the dipping motion, and two degrees in rotation to perform the operations to tilt and spin the forming tool (Figure S1 in the Supporting Information). The tilt degree of freedom was not used in this study. The setup consists of a translation stage (with a stepper motor Nema‐17) and two stepper motors (Sanyo Denki, 103H5208‐5210). System control was performed by using an Arduino Uno R3 microcontroller and three motor drivers (MotionKing, 2L415B). 3D‐printed elements were used to connect parts with each other. Donau drilling chucks were used to hold the carbon rods with PTFE tubes while dipping.

### Fabrication of the Variable‐Stiffness Thread (FRVST)

The fabrication of FRVSTs began with the preparation of a shape memory polymer (SMP) mixture using commercially available pellets of polyurethane (PU) polymer (MM5520, SMP Technologies). Pellets were dissolved in a solvent (dimethylformamide: DMF, Sigma‒Aldrich) in a weight proportion of 1 to 6 and stirred at 50 °C for 8 h. A polytetrafluoroethylene (PTFE) tube with an inner diameter of 1 mm and an outer diameter of 1.4 mm was dipped vertically in an already prepared SMP mixture using an on‐custom‐made dipping setup. Thereafter, the tube was hung vertically and cured in an oven at 80 °C for an hour. The dipping process was repeated one more time to obtain a 66 µm thick SMP layer. Then, a heating wire made of copper with a 0.1 mm diameter was wound around the SMP tube using an automated on‐custom‐made winding machine with a pitch distance of 1 mm. Afterward, the SMP tube with a heating wire was dipped 5 more times in the SMP mixture and cured in the oven. The sensing copper wire with a 0.052 mm thickness was wound around with a pitch distance of 1 mm. In the last fabrication step of the SMP tube, it was dipped into the SMP mixture to encapsulate the structure and achieve an external diameter of 2.3 mm. In parallel with SMP tube fabrication, a fluorocarbon wire with a 0.4 mm diameter was wound around the inner PTFE tube with a working channel and external diameters of 0.48 and 0.6 mm, respectively. The pitch distance in the cooling channel was 24 mm. Finally, the working channel with a helical channel was inserted into the SMP tube and glued from both sides. The silicone pipes were glued to the bottom part of the FRVST to provide cooling to the SMP layer and working channel.

### Three‐Point Flexural Test of the FRVST

To determine the SCF of the FRVSTs, a three‐point flexural test was conducted using a universal testing machine (Instron 5965) that had a temperature control box. The machine was equipped with 3D printed support parts made of acrylonitrile butadiene styrene (ABS) and a rail designed according to the ISO178:2019 standard that outlines the conditions for three‐point flexural tests on universal testing systems. The tests were performed at five temperatures, that was, 25 °C (room temperature) for the rigid state and 36, 40, 60, and 80 °C for the soft state, to obtain the deflection and reaction force of the FRVST. The raw data obtained from the reaction force against forced displacement were filtered using the Origin Pro 2016 infinite impulse response (IIR) filtering tool. A Butterworth filter with a pass frequency of 400 Hz was used for filtering.

### Characterization of the Heating and Cooling Times

For the characterization of the heating time, temperature readings were sampled using a thermal camera (FLIR, E8xt) that was placed 150 mm from the FRVST. Three samples with an SMP wall thickness of 0.45 mm were tested for each of the four applied voltages of 1.3, 1.7, 2, and 2.4 V, which resulted in applied powers of 1, 1.5, 2, and 2.5 W, respectively. The FRVST was cooled to room temperature between each measurement, and the heating profile of the FRVST was processed using FLIR ResearchIR software. For the characterization of the cooling time, the FRVST was heated to 80 °C at 2 W. After reaching the desired temperature, the heat was turned off, and active cooling was turned on. Active cooling was performed using an automated syringe pump (Figure S7 in the Supporting Information) under natural conditions (passive cooling) and three different rates equal to 0.03, 0.08, and 0.17 L mi^−1^n. All the cooling rates inside the FRVST were calculated using the parameters of the syringe pump, continuity law, and volume rate flow. For the cooling time characterization at different bending angles, the device was held in metallic grippers throughout the test.

### Surface Temperature Measurement

The surface temperature was measured using a previously presented method^[^ ^8^, ^19^ ^]^ by placing a TDK thermistor on the outer SMP surface while the temperature of the FRVST body was increased via indirect Joule heating. The FRVST was heated by applying 2 W with a power supply (BK Precision, 9141) when in air and 4.5 W when in water. The current was set to 1.5 A. Each of the applied powers resulted in a relative resistance change of 20% in the sensing wire. The sensing wire data were monitored with a digital multimeter (BK Precision, 5493C). The thermistor data were collected with data acquisition hardware (LabJack, U3‐HV).

### Actuation Stroke Angle Characterization of the Single‐Segment Catheter

*Reaction Time Demonstration of the Single‐Segment Catheter*: For the reaction time demonstration, the single‐segment catheter was placed upside down inside the aquarium in the middle of the working area of the eMNS. A repeatable magnetic field with a magnitude of 80 mT and shifting direction from 0 to 180° was applied.

### Multisegmented Catheter Demonstration

For the demonstration scenarios, the multisegmented catheter was placed upside down inside the aquarium in the middle of the working area of the eMNS. Each of the segments was stiffened and softened to demonstrate bending in different planes. A labyrinth demonstration was performed under the same conditions. Then, the aquarium was filled with human body temperature water. The water state was controlled with a thermometer. The catheter was inserted inside the 3D‐printed phantom of the human heart and omitted in water (Figure S8 in the Supporting Information). The heart was held in the 3D‐printed support. Blue and red waters were used to cool down the SMP layer and demonstrate the ablation procedure, respectively.

## Conflict of Interest

The authors have no conflicts of interest to declare.

## Supporting information

Supporting Information

Supplementary Video S1

Supplementary Video S2

Supplementary Video S3

Supplementary Video S4

Supplementary Video S5

Supplementary Video S6

Supplementary Video S7

## Data Availability

The data that support the findings of this study are available from the corresponding author upon reasonable request.

## References

[1] E. Bacha , D. Kalfa , Nat. Rev. Cardiol. 2014, 11, 24. 24189403 10.1038/nrcardio.2013.168

[2] A. Mosteiro , S. Amaro , R. Torné , L. Pedrosa , J. Hoyos , L. Llull , L. Reyes , A. Ferrés , N. de Riva , R. Mellado , J. Enseñat , Front. Neurol. 2022, 13, 884157. 35585845 10.3389/fneur.2022.884157 PMC9108381

[3] M. Han , L. Chen , K. Aras , C. Liang , X. Chen , H. Zhao , K. Li , N. R. Faye , B. Sun , J.‐H. Kim , W. Bai , Q. Yang , Y. Ma , W. Lu , E. Song , J. M. Baek , Y. Lee , C. Liu , J. B. Model , G. Yang , R. Ghaffari , Y. Huang , I. R. Efimov , J. A. Rogers , Nat. Biomed. Eng. 2020, 4, 997. 32895515 10.1038/s41551-020-00604-w PMC8021456

[4] S. S. Biere , M. I. Van Berge Henegouwen , K. W. Maas , L. Bonavina , C. Rosman , J. R. Garcia , S. S. Gisbertz , J. H. Klinkenbijl , M. W. Hollmann , E. S. De Lange , H. J. Bonjer , D. L. Van Der Peet , M. A. Cuesta , Lancet 2012, 379, 1887. 22552194 10.1016/S0140-6736(12)60516-9

[5] D. Neradi , V. Kumar , S. Kumar , P. Sodavarapu , V. Goni , S. S. Dhatt , Asian Spine J. 2022, 16, 279. 33966365 10.31616/asj.2020.0605 PMC9066260

[6] T. P. Martens , A. F. G. Godier , J. J. Parks , L. Q. Wan , M. S. Koeckert , G. M. Eng , B. I. Hudson , W. Sherman , G. Vunjak‐Novakovic , Cell Transplant. 2009, 18, 297. 19558778 10.3727/096368909788534915 PMC2771541

[7] T. J. Oxley , N. L. Opie , S. E. John , G. S. Rind , S. M. Ronayne , T. L. Wheeler , J. W. Judy , A. J. Mcdonald , A. Dornom , T. J. H. Lovell , C. Steward , D. J. Garrett , B. A. Moffat , E. H. Lui , N. Yassi , B. C. V. Campbell , Y. T. Wong , K. E. Fox , E. S. Nurse , I. E. Bennett , S. H. Bauquier , K. A. Liyanage , N. R. Van Der Nagel , P. Perucca , A. Ahnood , K. P. Gill , B. Yan , L. Churilov , C. R. French , P. M. Desmond , et al., Nat. Biotechnol. 2016, 34, 320. 26854476 10.1038/nbt.3428

[8] C. Chautems , A. Tonazzini , D. Floreano , B. J. Nelson , presented at 2017 IEEE/RSJ Int. Conf. Intelligent Robots Systems (IROS) , Vancouver, BC, Canada, Dec. 2017.

[9] N. R. Grubb , BMJ 2001, 322, 777. 11282867 10.1136/bmj.322.7289.777 PMC1119956

[10] Z. Li , L. Wu , H. Ren , H. Yu , Mech. Mach. Theory 2017, 107, 148.

[11] C. Pappone , G. Vicedomini , F. Manguso , F. Gugliotta , P. Mazzone , S. Gulletta , N. Sora , S. Sala , A. Marzi , G. Augello , L. Livolsi , A. Santagostino , V. Santinelli , J. Am. Coll. Cardiol. 2006, 47, 1390. 16580527 10.1016/j.jacc.2005.11.058

[12] E. S. Gang , B. L. Nguyen , Y. Shachar , L. Farkas , L. Farkas , B. Marx , D. Johnson , M. C. Fishbein , C. Gaudio , S. J. Kim , Circ. Arrhythmia Electrophysiol. 2011, 4, 770. 10.1161/CIRCEP.110.959692 21690463

[13] M. Kawamura , M. M. Scheinman , Z. H. Tseng , B. K. Lee , G. M. Marcus , N. Badhwar , J. Interventional Card. Electrophysiol. 2017, 48, 35. 10.1007/s10840-016-0158-x 27314679

[14] C. Chautems , A. Tonazzini , Q. Boehler , S. H. Jeong , D. Floreano , B. J. Nelson , Adv. Intel. Syst. 2020, 2, 1900086.

[15] J. K.‐R. Chun , S. Ernst , S. Matthews , B. Schmidt , D. Bansch , S. Boczor , A. Ujeyl , M. Antz , F. Ouyang , K.‐H. Kuck , Eur. Heart J. 2007, 28, 190. 17218451 10.1093/eurheartj/ehl447

[16] C. Piorkowski , C. Eitel , S. Rolf , K. Bode , P. Sommer , T. Gaspar , S. Kircher , U. Wetzel , A. S. Parwani , L.‐H. Boldt , M. Mende , A. Bollmann , D. Husser , N. Dagres , M. Esato , A. Arya , W. Haverkamp , G. Hindricks , Circ. Arrhythmia Electrophysiol. 2011, 4, 157. 10.1161/CIRCEP.110.957761 21248246

[17] P. Kanagaratnam , M. Koa‐Wing , D. T. Wallace , A. S. Goldenberg , N. S. Peters , D. W. Davies , J. Interventional Card. Electrophysiol. 2008, 21, 19. 10.1007/s10840-007-9184-z PMC2262917 18202905

[18] L. Mantziari , I. Suman‐Horduna , M. Gujic , D. G. Jones , T. Wong , V. Markides , J. P. Foran , S. Ernst , Pac. Clin. Electrophysiol. 2013, 36, 757. 10.1111/pace.12113 23438182

[19] Y. Piskarev , J. Shintake , C. Chautems , J. Lussi , Q. Boehler , B. J. Nelson , D. Floreano , Adv. Funct. Mater. 2022, 32, 2107662.

[20] J. Lussi , M. Mattmann , S. Sevim , F. Grigis , C. De Marco , C. Chautems , S. Pané , J. Puigmart‐Luis , Q. Boehler , B. J. Nelson , Adv. Sci. 2021, 8, 2101290. 10.1002/advs.202101290 PMC8456283 34272935

[21] M. Mattmann , C. De Marco , F. Briatico , S. Tagliabue , A. Colusso , X.‐Z. Chen , J. Lussi , C. Chautems , S. Pané , B. Nelson , Adv. Sci. 2022, 9, 2103277. 10.1002/advs.202103277 PMC8728812 34723442

[22] M. Brancadoro , M. Manti , F. Grani , S. Tognarelli , A. Menciassi , M. Cianchetti , Front. Robot. AI 2019, 6, 12. 33501028 10.3389/frobt.2019.00012 PMC7806054

[23] M. Cianchetti , T. Ranzani , G. Gerboni , T. Nanayakkara , K. Althoefer , P. Dasgupta , A. Menciassi , Soft Rob. 2014, 1, 122.

[24] Y.‐J. Kim , S. Cheng , S. Kim , K. Iagnemma , IEEE Trans. Robot. 2013, 29, 1031.

[25] T. P. Chenal , J. C. Case , J. Paik , R. K. Kramer , presented at 2014 IEEE/RSJ Int. Conf. Intelligent Robots Systems , Chicago, IL, USA, Nov. 2014.

[26] A. Tonazzini , S. Mintchev , B. Schubert , B. Mazzolai , J. Shintake , D. Floreano , Adv. Mater. 2016, 28, 10142. 27689347 10.1002/adma.201602580

[27] R. Zhao , Y. Yao , Y. Luo , J. Med. Dev. 2016, 10, 021002.

[28] Q. Gao , Z. Sun , Actuators 2021, 10, 130.

[29] H. M. Le , P. T. Phan , C. Lin , L. Jiajun , S. J. Phee , Ann. Biomed. Eng. 2020, 48, 1837. 32232695 10.1007/s10439-020-02495-z

[30] B. Mazzolai , A. Mondini , E. del Dottore , L. Margheri , F. Carpi , K. Suzumori , M. Cianchetti , T. Speck , S. K. Smoukov , I. Burgert , T. Keplinger , G. D. F. Siqueira , F. Vanneste , O. Goury , C. Duriez , T. Nanayakkara , B. Vanderborght , J. Brancart , S. Terryn , S. I. Rich , R. Liu , K. Fukuda , T. Someya , M. Calisti , C. Laschi , W. Sun , G. Wang , L. Wen , R. Baines , et al., Multifunct. Mater. 2022, 5, 032001.

[31] V. Kanyanta , A. Ivankovic , J. Mech. Behav. Biomed. Mater. 2010, 3, 51. 19878902 10.1016/j.jmbbm.2009.03.005

[32] A. P. S. Selvadurai , J. Mech. Phys. Solids 2006, 54, 1093.

[33] H. Lv , J. Leng , Y. Liu , S. Du , Adv. Eng. Mater. 2008, 10, 592.

[34] M. Raja , S. H. Ryu , A. M. Shanmugharaj , Eur. Polym. J. 2013, 49, 3492.

[35] B. Aksoy , H. Shea , Adv. Funct. Mater. 2020, 30, 2001597.

[36] P. Prathumrat , S. Tiptipakorn , S. Rimdusit , Smart Mater. Struct. 2017, 26, 065025.

[37] J. Delaey , P. Dubruel , S. Van Vlierberghe , Adv. Funct. Mater. 2020, 30, 1909047.

[38] Y.‐J. Wang , U.‐S. Jeng , S.‐H. Hsu , ACS Biomater. Sci. Eng. 2018, 4, 1397. 33418669 10.1021/acsbiomaterials.8b00091

[39] M. Cabanlit , D. Maitland , T. Wilson , S. Simon , T. Wun , M. E. Gershwin , J. Van De Water , Macromol. Biosci. 2007, 7, 48. 17238230 10.1002/mabi.200600177

[40] W. Small Iv , T. S. Wilson , W. J. Benett , J. M. Loge , D. J. Maitland , Opt. Express 2005, 13, 8204. 19498850 10.1364/opex.13.008204

[41] S. Farè , V. Valtulina , P. Petrini , E. Alessandrini , G. Pietrocola , M. C. Tanzi , P. Speziale , L. Visai , J. Biomed. Mater. Res. A 2005, 73, 1. 15704114 10.1002/jbm.a.30193

[42] F. Keçe , K. Zeppenfeld , S. A. Trines , Arrhythmia Electrophysiol. Rev. 2018, 7, 169. 10.15420/aer.2018.7.3 PMC6141930 30416730

[43] M. Houmsse , E. G. Daoud , Expert Rev. Med. Dev. 2012, 9, 59. 10.1586/erd.11.42 22145841

[44] H.‐W. Fang , K.‐Y. Li , T.‐L. Su , T. C.‐K. Yang , J.‐S. Chang , P.‐L. Lin , W.‐C. Chang , Mater. Lett. 2008, 62, 3739.

[45] H. Nakagawa , W. S. Yamanashi , J. V. Pitha , M. Arruda , X. Wang , K. Ohtomo , K. J. Beckman , J. H. Mcclelland , R. Lazzara , W. M. Jackman , Circulation 1995, 91, 2264. 7697856 10.1161/01.cir.91.8.2264

[46] L. Blanc , A. Delchambre , P. Lambert , Actuators 2017, 6, 23.

[47] E. Nof , W. G. Stevenson , R. M. John , Arrhythmia Electrophysiol. Rev. 2013, 2, 45. 10.15420/aer.2013.2.1.45 PMC4711562 26835040

[48] T. L. Buckner , R. Kramer‐Bottiglio , Multifunct. Mater. 2018, 1, 012001.

[49] M. C. Yuen , R. A. Bilodeau , R. K. Kramer , IEEE Robot. Automation Lett. 2016, 1, 708.

[50] R. Yu , S. L. Charreyron , Q. Boehler , C. Weibel , C. Chautems , C. C. Poon , B. J. Nelson , presented at 2020 IEEE Int. Conf. Robotics Automation (ICRA) , Paris, France, Sept. 2020.

[51] M. S. Xavier , A. J. Fleming , Y. K. Yong , Adv. Intel. Syst. 2021, 3, 2000187.

[52] J. Shintake , B. Schubert , S. Rosset , H. Shea , D. Floreano , presented at 2015 IEEE/RSJ Int. Conf. Intelligent Robots Systems (IROS) , Hamburg, Germany, Dec. 2015.

[53] Y. Piskarev , J. Shintake , V. Ramachandran , N. Baugh , M. D. Dickey , D. Floreano , Adv. Intel. Syst. 2020, 2, 2000069.

[54] C. Tangwongsan , J. A. Will , J. G. Webster , K. L. Meredith , D. M. Mahvi , IEEE Trans. Biomed. Eng. 2004, 51, 1478. 15311835 10.1109/TBME.2004.828035

[55] C. Tangwongsan , L. Chachati , J. G. Webster , P. V. Farrell , Biomed. Eng. Online 2006, 5, 57. 17067386 10.1186/1475-925X-5-57 PMC1635717

[56] C. Tangwongsan , presented at 2007 IEEE/NIH Life Science Systems Applications Workshop, Bethesda, MD, USA, Dec. 2007.

[57] M. Mattmann , Q. Boehler , X. Chen , S. Pané , B. J. Nelson , presented at 2012 IEEE/RSJ Int. Conf. Intelligent Robots Systems (IROS) , Kyoto, Japan, 2022.

[58] W. Ullah , R. J. Schilling , T. Wong , J. Atr. Fibrillation 2016, 8, 1282. 27909471 10.4022/jafib.1282 PMC5089484

[59] F. Bessière , C. Zikry , L. Rivard , K. Dyrda , P. Khairy , Europace 2018, 20. 10.1093/europace/euy006 29722859

[60] L. Capron , P. Bruneval , Cardiovasc. Res. 1989, 23, 941. 2611802 10.1093/cvr/23.11.941

[61] K. Takashima , R. Shimomura , T. Kitou , H. Terada , K. Yoshinaka , K. Ikeuchi , Tribol. Int. 2007, 40, 319.

